# A new subtype of eastern tick-borne encephalitis virus discovered in Qinghai-Tibet Plateau, China

**DOI:** 10.1038/s41426-018-0081-6

**Published:** 2018-04-25

**Authors:** Xiaoyi Dai, Guobao Shang, Shan Lu, Jing Yang, Jianguo Xu

**Affiliations:** 1 State Key Laboratory for Infectious Disease Prevention and Control, National Institute for Communicable Disease Control and Prevention, Collaborative Innovation Center for Diagnosis and Treatment of Infectious Diseases, Chinese Center for Disease Control and Prevention, 102206 Beijing, Changping China; 2 Haixi Prefecture Center for Disease Control and Prevention, 817000 Haixi Prefecture, Qinghai China; 3 Shanghai Institute for Emerging and Re-emerging infectious diseases, Shanghai Public Health Clinical Center, 201508 Shanghai, Jinshan China

## Abstract

Tick-borne encephalitis virus (TBEV) has been classified into three subtypes, namely the European (Eu-TBEV), Far Eastern (FE-TBEV), and Siberian (Sib-TBEV). In this study, we discovered a new subtype of TBEV in wild rodent *Marmota himalayana* in Qinghai-Tibet Plateau in China, proposed as subtype Himalayan (Him-TBEV). Two complete genomes of TBEV were obtained from respiratory samples of 200 marmots. The phylogenetic analysis using the E protein and polyprotein demonstrated that the two strains of Him-TBEV formed an independent branch, separated from Eu-TBEV, Sib-TBEV, and FE-TBEV. The nomenclature of Him-TBEV as a new subtype was also supported by comparative analysis using nucleotide and amino acid sequences of E protein and polyprotein. For E protein, The Him-TBEV showed 82.6–84.6% nucleotide identities and 92.7–95.0% amino acid identities with other three subtypes. For polyprotein, the Him-TBEV showed 83.5–85.2% nucleotide identities and 92.6–94.2% amino acids identities with other three subtypes. Furthermore, of 69 amino acid substitutions profiles detected in complete polyprotein of 112 strains of TBEV, Him-TBEV subtype displayed unique amino acids in the 36 positions. Notably, for the subtype-specific amino acid position 206 of E protein, Him-TBEV shared the Val with Eu-TBEV, but differed from FE-TBEV and Sib-TBEV. The evolutionary analysis with BEAST suggested that Him-TBEV diverged from other subtypes of eastern TBEV group about 2469 years ago. It should be mentioned that Qinghai-Tibet Plateau in China is the plague endemic region where *Marmota himalayana* is the primary host. The public health significance of discovery of Him-TBEV in *Marmota himalayana* must be carefully evaluated.

## Introduction

Tick-borne encephalitis (TBE) is a severe central nervous system infection caused by tick-borne encephalitis virus (TBEV). Over 10,000 human infections have been reported annually in endemic regions of Eurasian continent, especially in Russia.^1–3^ The causative agent TBEV is a member of genus *Flavivirus*, family *Flaviviridae*. The virus genome is a positive single-stranded RNA molecule, approximately 11,000 bases in length, which encodes three structural (capsid, C; membrane, M, which is expressed as precursor prM; envelope, E) and seven non-structural proteins (NS1, NS2A, NS2B, NS3, NS4A, NS4B, and NS5).^4^ Based on phylogenetic analysis of E protein, the TBEV has been classified into three subtypes, namely European (Eu-TBEV), Far-Eastern (FE-TBEV), and Siberian (Sib-TBEV).^5, 6^ The Sib-TBEV and FE-TBEV were also called eastern TBEV. Recently, 886-84-like strains were divided from Sib-TBEV and proposed as a new subtype named Baikalian (Bkl-TBEV).^7^ Members of these three subtypes showed difference in geographical distribution, virulence, clinical severity, and the difference in the nucleotide (14.6–16.5%) and amino acid (5.0–6.8%) sequences of their polyprotein.^8^ In China, the first human infection was traced back to 1943, and the strains of TBEV were first isolated from patients and ticks in 1952.^9, 10^ Recently, the number of human infections in China has increased notably. A total of 2117 human infections were reported in the period from 2006 to 2013. Two subtypes of TBEV had been identified in China, namely FE-TBEV and Sib-TBEV. The FE-TBEV was widely detected in several provinces, such as Heilongjiang, Jilin, Liaoning, Xinjiang, Yunnan, and Tibet. The Sib-TBEV was only detected in Xinjiang.^11–14^ Furthermore, TBEV has been detected in previously unaffected areas,^3, 12, 15^ making the disease a growing public health threat. In this study, we discovered two new strains of TBEV in wild rodent *Marmota himalayana* in Qinghai-Tibet Plateau in China, which represented a new subtype that had been concealed for hundred years.

## Results

### The respiratory virome of *Marmota himalayana*

Only 66 contigs with a length range of 125–7419 nt were assembled from RNA-seq sequencing showing similarity to RNA viral sequences. These contigs could be classified into six RNA virus families and one RNA virus genus: *Flaviviridae* (*n* = 27)*, Phenuiviridae* (*n* = 8)*, Picornaviridae* (*n* = 3)*, Picobirnaviridae* (*n* = 20)*, Dicistroviridae* (*n* = 1)*, Narnaviridae* (*n* = 3) and *Sobemovirus* (*n* = 4). Importantly, 3 contigs with lengths of 2965 nt, 4647 nt and 7419 nt showed 99% amino acid identity to *Marmota Himalayana* hepatovirus (MHHAV) within family *Picornaviridae* which has been reported by Yu.^16^ Contigs with a length range of 125–775 nt showed 89.1–100% amino acid identities to different strains of tick-borne encephalitis virus (TBEV) within family *Flaviviridae*.

### Discovery of genomes of TBEV from *Marmota himalayana*

The existence of TBEV in *Marmota himalayana* was revealed by RNA-seq sequencing. Two TBEV-positive low respiratory tissues were detected by RT-PCR method. The complete genome sequence data for two strains of TBEV from *Marmota himalayana* in the present study were obtained by combination method of RNA-seq sequencing and RT-PCR products amplified by a set of primers covering the whole genome region. The two genomes shared the same length of 10,761 nucleotides, with 99.5% identity. The complete genome sequences were submitted to the GenBank with accession numbers MG599476 and MG599477. The single open reading frame (ORF) of the genome with 10,245-nt in length that encodes three structural proteins (C, prM, and E) and 7 nonstructural proteins (NS1, NS2A, NS2B, NS3, NS4A, NS4B and NS5) and was flanked by two non-coding regions (NCR), with a 132-nt NCR at 5′ end and a 384-nt NCR at 3′ end. The genome organization and sequence analysis identified the two genomes as members of TBEV, which were named as Himalaya-1 and Himalaya-2, respectively.

### Genome analysis

The nucleotide sequences of the E protein and polyprotein were used to reveal the phylogenetic relationship of the Himalaya-1 and Himalaya-2 with the members of other TBEV lineages. The complete nucleotide sequences of E protein (1488 nt) and polyprotein (10,245 nt) of 23 virus genomes were included in the study, including 7 strains of FE-TBEV, 4 strains of Sib-TBEV, 3 strains of Eu-TBEV, 1 strain of Baikalian subtype and TBEV strain 178–79. In addition, 4 strains from animals in European countries were also included, such as Turkish sheep encephalitis virus (TSEV), Greek goat encephalitis virus (GGEV), Louping ill virus (LIV) and Spanish sheep encephalitis virus (SSEV), which closely related to Eu-TBEV.

**Fig. 1:**
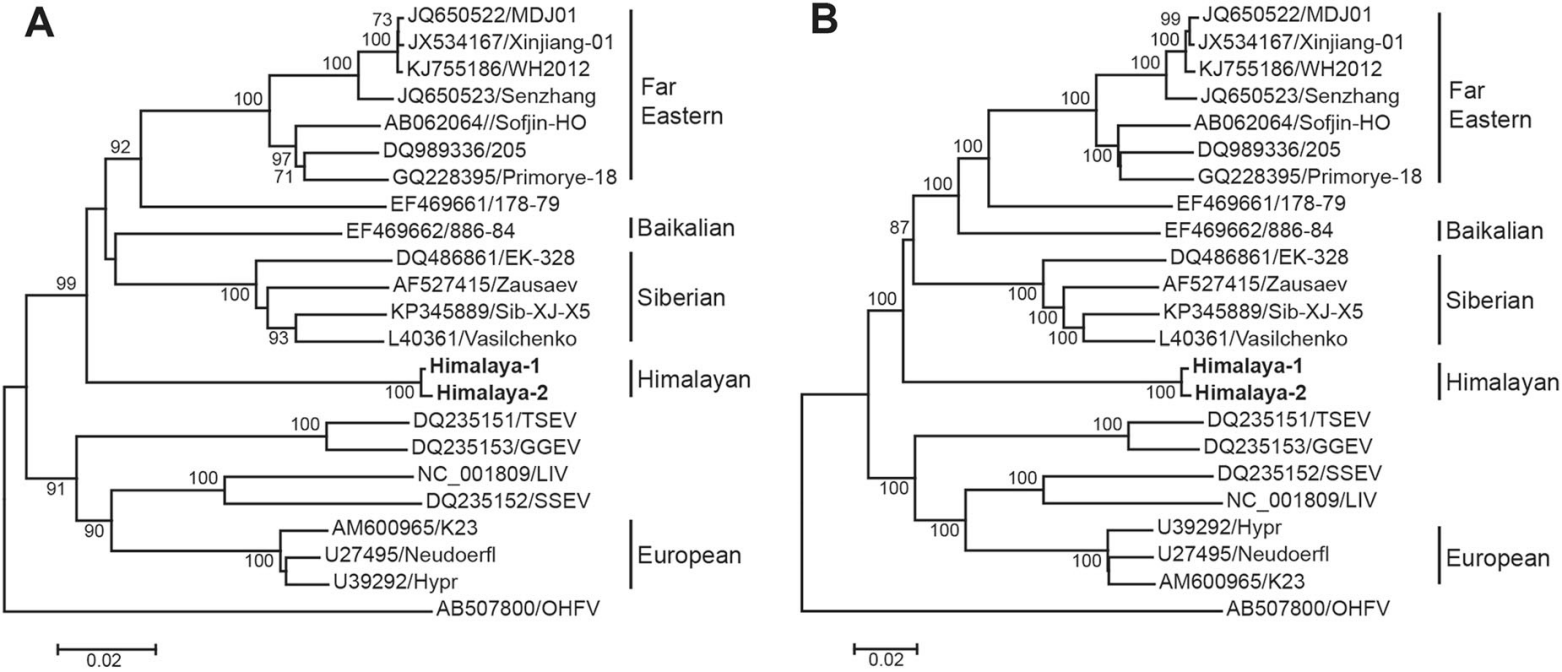
Phylogenetic relationship of Himalayan subtype with other subtypes of TBEV. **a** The phylogenetic tree of E protein. **b** The phylogenetic tree of polyprotein. Strains of Him-TBEV were indicated in boldface. Phylogenetic trees were constructed using MEGA 6.0 with neighbor-joining method (1000 bootstrap replications). Bootstrap values (>70%) are shown at the branches. Scale bar below indicates the nucleotide substitutions per site

Phylogenetic trees were constructed by the neighbor-joining (NJ) method with 1000 bootstrap replications. For phylogenetic tree of E protein (Fig. 1a), Omsk hemorrhagic fever virus (OHFV) was used as outgroup, the TBEV strains were formed two clades. One clade included Eu-TBEV, TSEV, GGEV, LIV, and SSEV, which discovered from western European countries. Another clade included eastern TBEV group (FE-TBEV, Sib-TBEV and Bkl-TBEV), Himalaya-1 and Himalaya-2. Clearly, the two TBEV strains from *Marmota himalayana* formed a separate lineage, from where the FE-TBEV and Bkl-TBEV were further diverged.

When the complete nucleotide sequences of polyprotein (10,245 nt) from the same set of strains were used to construct the phylogenetic tree with same method, the same topology was obtained (Fig. 1b). These results strongly suggested that the Himalaya-1 and Himalaya-2 are indeed representing a new subtype of TBEV, which named Himalayan subtype (Him-TBEV), differed from FE-TBEV, Sib-TBEV, and Eu-TBEV as well.

We then conducted comparative analysis of E protein and polyprotein of between and within the subtypes. The nucleotide and amino acid identities of E protein of Himalaya-1 and Himalaya-2 were both of 99.6%. The nucleotide and amino acid identities of polyprotein between Himalaya-1 and Himalaya-2 were 99.5 and 99.8%, respectively (Supplementary Table S1). For polyprotein, the Him-TBEV showed 83.5–85.2% nucleotide identities and 92.6–94.2% amino acid identities with other three subtypes; the identity level of nucleotide and amino acid among Eu-TBEV, Sib-TBEV, and FE-TBEV subtypes were 83.2%–85.5% and 93.0%–95.2%, respectively. For E protein, the Him-TBEV shared 82.6–84.6% nucleotide identities and 92.7–95.0% amino acid identities with other three subtypes; the identity level of nucleotide and amino acid among the Eu-TBEV, Sib-TBEV, and FE-TBEV subtypes were 82.9%–85.7% and 95.2%–98.0%, respectively (Supplementary Table S1).

### The unique signature amino acid sequence profile of Him-TBEV

The published genomes from a total of 112 strains of TBEV currently available in GenBank were downloaded for analysis, including 60 strains of FE-TBEV, 22 strains of Sib-TBEV and 30 strains of Eu-TBEV (Supplementary Table S2).

Notably, the analysis with deduced amino acid sequences of Himalaya-1 and Himalaya-2 revealed 69 amino acid substitutions in the complete polyprotein (Table 1). Most of these substitutions were located at E, NS1, NS2A, and NS4B proteins. No substitutions were found in the well-known features of E protein, such as fusion peptide, tick-specific peptide EHLPTA, 12 cysteine residues.^17^ Three substitutions (positions 16, 143, and 151) were located in domain I, two substitutions (positions 130 and 201) were located in domain II and three substitutions (positions 331, 346 and 349) were located in domain III of E protein.^18^ The substitution of amino acid position 201 of E protein (E → A) would increase the net positive charge of the virus surface, as with mutant (E → K) described previously^19^.

**Table 1:** Amino acid substitutions between Himalayan subtype and other three subtypes^a^

Amino acid position	TBEV subtypes				Amino acid	TBEV subtypes			
	Himalayan	Far Eastern	Siberian	European	position	Himalayan	Far Eastern	Siberian	European
C-4	**E**	**K**	**K**	**K**	NS2A-106	**T**	**M**	**M**	**M**
C-50	M	V/I	I	V	NS2A-150	**R**	**K**	**K**	**K**
C-84	I	T/S	T/A/S	A	NS2A-171	Q	Y/H	Y/H/C	H/R
prM-4	**T**	**A**	**A**	**A**	NS2A-182	S	C	A/V	A
prM-12	**T**	**D**	**D**	**D**	NS2A-184	**A**	**S**	**S**	**S**
prM-14	I	T/P	T	S	NS2A-208	I	L	M/L/V	L
prM-94	**A**	**S**	**S**	**S**	NS2B-20	**I**	**M**	**M**	**M**
prM-101	V	A/P	A	A	NS2B-94	T	V	V/A/M	M
prM-103	**R**	**G**	**G**	**G**	NS2B-104	V	A	A/S	A
prM-154	A	V/T/I	V	V	NS2B-127	**K**	**R**	**R**	**R**
E-16	H	Q	Q/R	Q	NS3-36	**H**	**Q**	**Q**	**Q**
E-130	**Y**	**H**	**H**	**H**	NS3-105	**E**	**G**	**G**	**G**
E-143	**I**	**V**	**V**	**V**	NS3-160	**S**	**N**	**N**	**N**
E-151	**I**	**V**	**V**	**V**	NS3-182	**V**	**A**	**A**	**A**
E-201	A	E	E	E/K	NS3-254	T	G	G/S	S
E-331	E	A	T	T/S	NS3-405	E	D/N	D	D
E-346	**T**	**A**	**A**	**A**	NS3-440	**I**	**V**	**V**	**V**
E-349	I	S	S/F	S/F	NS3-593	**K**	**T**	**T**	**T**
E-452	**M**	**V**	**V**	**V**	NS4A-110	**V**	**I**	**I**	**I**
E-462	L	A/M/V	V	V	NS4B-16	I	V	V	V/A
E-480	**L**	**F**	**F**	**F**	NS4B-17	M	L/S	L	L
E-486	**V**	**L**	**L**	**L**	NS4B-22	K	E	E/D	E/G
NS1-49	M	A/T	T	T	NS4B-135	**K**	**R**	**R**	**R**
NS1-50	**Y**	**F**	**F**	**F**	NS4B-175	**A**	**V**	**V**	**V**
NS1-73	**V**	**T**	**T**	**T**	NS4B-183	T	M	M	M
NS1-105	**R**	**L**	**L**	**L**	NS4B-191	L	V	V	V/M
NS1-124	**V**	**I**	**I**	**I**	NS4B-198	G	S/A	S/L	S
NS1-147	**G**	**E**	**E**	**E**	NS4B-214	**E**	**D**	**D**	**D**
NS1-175	T	S/P/L	P/A	P/S	NS4B-216	V	L/Q	L	L
NS1-228	**S**	**A**	**A**	**A**	NS5-45	G	R/K	R/K	R
NS1-277	A	T/I	T/V	I	NS5-335	**S**	**A**	**A**	**A**
NS1-290	**T**	**A**	**A**	**A**	NS5-445	V	A/T	A	M
NS2A-50	F	L	M	M	NS5-511	T	I/V	I	I
NS2A-52	E	K/R	K	R	NS5-699	T	V/A	V/A	V/A/P
NS2A-54	E	K/R	K	R					

^a^TBEV strains used for polyprotein alignment are listed in Supplementary Table S2. The unique amino acid substitutions of Him-TBEV are shown in boldface

Of 69 amino acid substitutions observed in the complete polyprotein, Him-TBEV has 36 unique substitutions. At these 36 positions, the amino acids were conserved in all other three subtypes, showing identical amino acids. However, Him-TBEV displayed unique amino acids in the 36 positions (Table 1). These data strongly supported the hypothesis that the Him-TBEV is indeed a new subtype of TBEV. Interestingly, for the so-called subtype-specific amino acid position 206 of E protein, Him-TBEV shared the Val with that of Eu-TBEV, but differed from that of FE-TBEV and Sib-TBEV.^5^

Of the 17 pathogenicity associated amino acid residues, the Him-TBEV shared 9 substitutions that are specific to pathogenic strains, and 5 substitutions that are specific to strains from patients with the subclinical presentation. These pathogenicity associated amino acid residues were proposed by analyzing the polyprotein of pathogenic strains isolated from the brains of dead patients with the encephalitis, from the blood of patients with febrile, and from patients with subclinical symptoms.^20, 21^ In short, the profile of pathogenic associated amino acid substitution of Him-TBEV is similar to low virulence strain Oshima 5–10 (Table 2).

**Table 2:** 17 key amino acid positions associated with virulence of TBEV strains

Amino acid positions	TBEV strains							
	Senzhang	Sofijin	Shkotovo	Oshima	Pry-253	Pry-270	Him1	Him2
C-32	Q	Q	Q	R	R	R	Q	Q
C-69	K	K	K	K	R	R	K	K
C-100	D	D	D	D	N	N	D	D
C-111	V	L	V	V	Del	Del	V	V
PrM-155	A	A	A	V	V	V	A	A
E-463	V	V	V	A	A	A	A	A
NS1-141	S	S	S	S	G	G	G	G
NS2B-108	F	F	F	F	V	V	F	F
NS3-16	R	R	K	R	K	K	R	R
NS3-45	S	S	F	F	F	F	S	S
NS4B-95	M	M	M	M	V	V	V	V
NS4B-179	V	V	V	V	A	A	V	V
NS4B-213	A	A	A	A	V	V	V	V
NS5-634	S	S	S	T	T	T	A	A
NS5-677	G	G	G	G	K	K	R	R
NS5-692	I	I	I	V	V	V	V	V
NS5-724	A	A	A	A	S	S	V	V

Sofijin-HO, Sofijin; Shkotovo 94, Shkotovo; Oshima 5-10, Oshima; Primorye-253, Pry-253; Primorye-270, Pry-270; Himalaya-1, Him1; Himalaya-2, Him2.

The gray shaded amino acids signify the amino acids in and as identical with strain of Senzhang

### Estimated divergence time of Him-TBEV

**Fig. 2:**
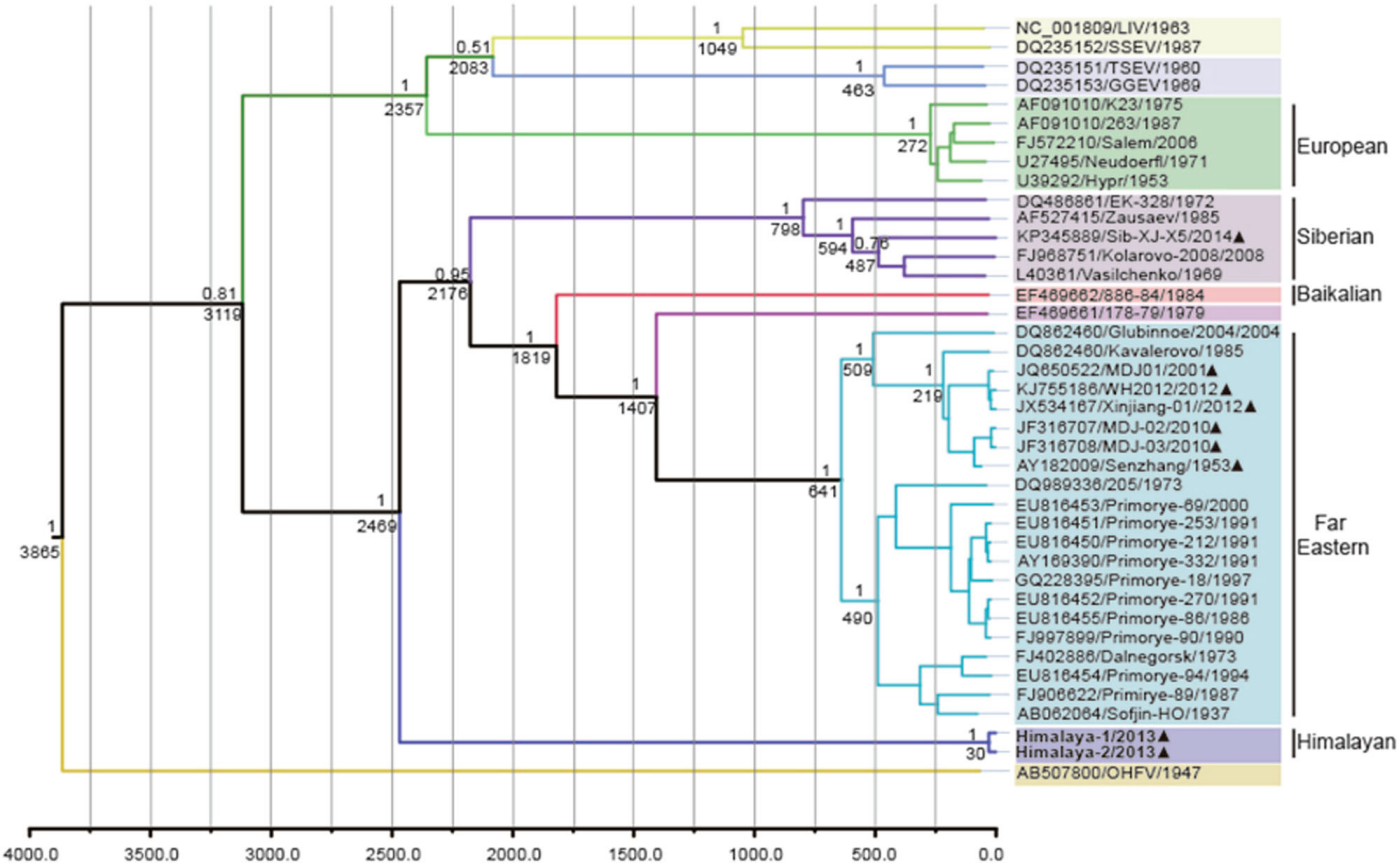
The Bayesian maximum clade credibility tree based on nucleotide sequences of E protein. The year of sampling, strain name and accession number are on the tip labels. Node labels indicate the most recent common ancestor (tMRCA) and the posterior probabilities. Strains of Him-TBEV were indicated in boldface. The TBEV strains detected in China were marked with black triangle

To understand the temporal constraints on the origin and dispersal of the TBEV group, BEAST was used to estimate the time of the most recent common ancestor (tMRCA) of each lineage, based on nucleotide sequences of E protein (1488 nt). Maximum clade credibility (MCC) tree was constructed with 40 representative strains of TBEV and related flaviviruses, including 5strains of Eu-TBEV, 5 strains of Sib-TBEV, 21 strains of FE-TBEV, 1 strain of Baikalian subtype and TBEV strain 178–79. The resulting tree is shown in Fig. 2. The most recent common ancestor of the Him-TBEV and the other subtypes of TBEV was estimated about 3119 (95% HPD = 1123–7695) years ago, when the western TBEV group (Eu-TBEV) and the eastern TBEV group were diverged. The Him-TBEV was first diverged with the eastern TBEV group about 2469 (95% HPD = 956–6376) years ago. The Sib-TBEV was formed about 800 years ago. The Baikalian subtype was diverged about 1820 years ago. Most nodes had 100% bootstrap support in the neighbor-joining tree. It should be mentioned that Chinese isolates belong to two subtypes. The strain of Sib-TBEV (Sib-XJ-X5) emerged about 480 years ago. The strains of FE-TBEV in china emerged about 210 years ago, which widely distributed the northeastern region of China (Fig. 2).

## Discussion

We have discovered two novel strains (Himalaya-1 and Himalaya-2) of TBEV in *Marmota himalayana* for the first time in Qinghai-Tibet Plateau, China. Phylogenetic analysis, which has been widely used to study the genetic population of TBEV, clearly suggested that the Him-TBEV separated from other three subtypes (FE-TBEV, Sib-TBEV, and Eu-TBEV). The topology of phylogenetic trees demonstrated that the Him-TBEV could be considered as a new member of eastern TBEV group, together with FE-TBEV and Sib-TBEV (Fig. 1). Classification of the Him-TBEV was also supported by comparative analysis of E protein and polyprotein. At amino acid level, the diversity of the E protein was less than 2.2% within the subtype and 3.6–5.6% between the subtypes, which is in the range of variation reported for other flaviviruses (3–10%).^5, 22–25^ Him-TBEV differed by 5.0–7.3% from the other subtypes. The same was observed for the polyprotein where 4.8–7.4% divergence calculated between Him-TBEV and the other subtypes was almost identical to the value 5.0–6.8%^8^, the range of distances found between conventional three subtypes (Supplementary Table S1).

Classification of Him-TBEV as a new subtype was further supported by signature amino acid analysis. Notably, 69 amino acid substitutions in complete polyprotein were found in comparison with 112 strains of the three subtypes, of which 36 substitutions were unique for Him-TBEV, but the amino acids substitution in the same position were conserved in the three subtypes (Table 1). For the so-called subtype-specific amino acid position 206 of E protein, Him-TBEV shared the Val with that of Eu-TBEV, but differed from that of FE-TBEV and Sib-TBEV, which suggested that the amino acid at the 206 position were not unique between subtypes of TBEV.^5^

Although the classification of TBEV is mainly based on phylogenetic analysis and differences in nucleotide and amino acid sequences of E protein and polyprotein, isolation of a strain is required for full recognition of Him-TBEV as a new subtype.

With the linear and nonlinear regression analysis of genetic versus geographic distance combined with BEAST analysis, Heinze et al. reported that the most recent common ancestor of the TBEV group was present at 3119 year ago^26^. When Him-TBEV was included and analyzed with similar methodology, it was observed that the Him-TBEV and the eastern TBEV group were diverged about 2469 years ago. After the divergence, the Sib-TBEV was separated from the FE-TBEV subtype about 2176 years ago (Fig. 2).

**Fig. 3:**
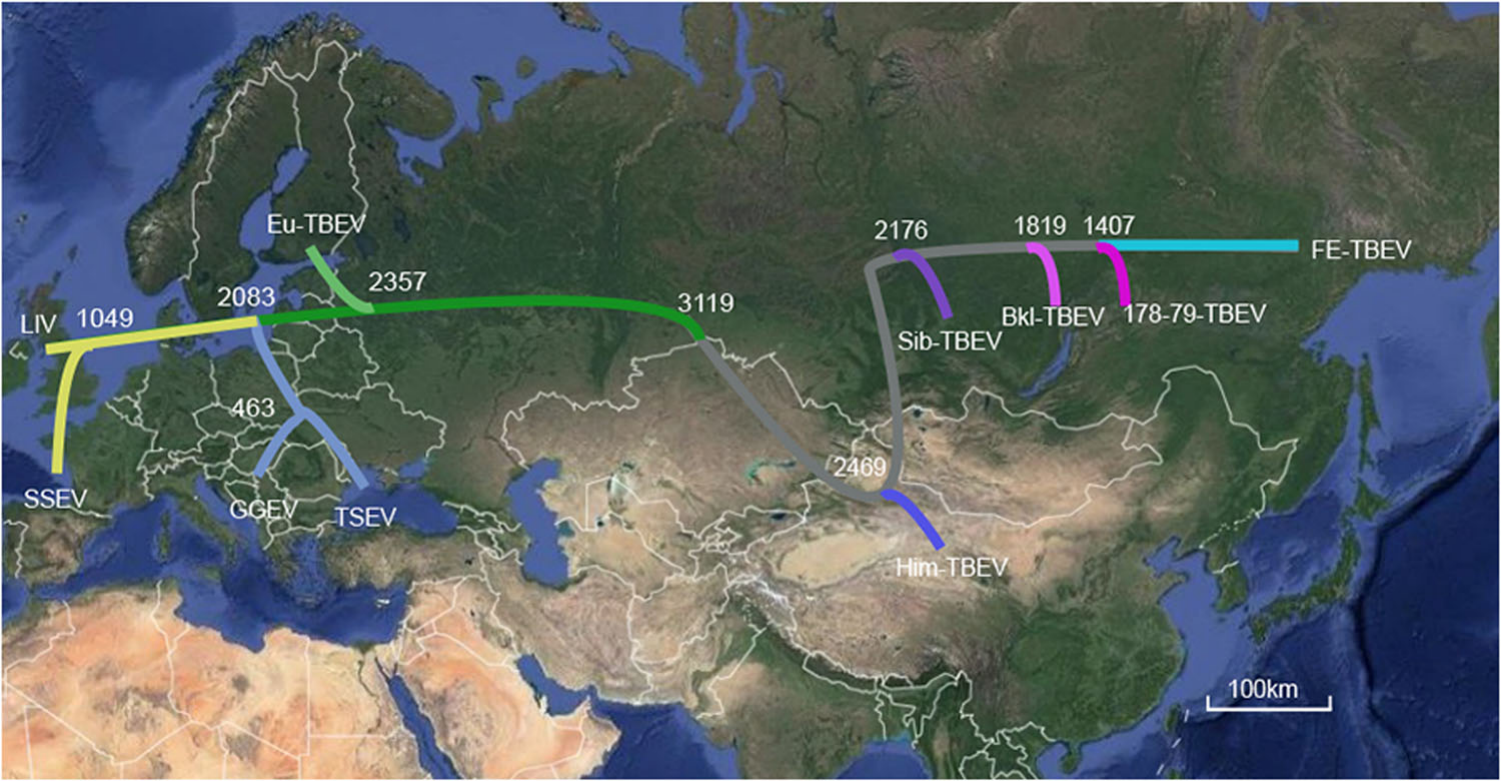
A possible model of TBEV dispersal. The maximum clade credibility tree is plotted onto geographic location. The time of estimated tMRCA of each phylogenetic linage are indicated at the major nodes in the tree

The topology of our MCC tree was almost consistent with that reported by Heinze et al.^26^. Those results suggested that Him-TBEV is a very ancient lineage of eastern TBEV (Figs. 2, 3). When geographic site was considered, it is possible that after divergence with western TBEV, the Him-TBEV was separated with rest eastern TBEV subtypes about 2469 years ago, in Himalaya region (Fig. 3). The FE-TBEV and Sib-TBEV were then progressed eastwards to Russian, and northeastern China.

It should be mentioned that the Qinghai-Tibet Plateau is the highest and one of the most extensive plateaus in the world, covering an area of 2.5 × 10^6^ km^2^ with an average elevation of more than 4000 m. Qinghai-Tibetan Plateau of China is known to be the plague endemic region where marmot *Marmota himalayana* is the primary host^27^. Most human-inhabited areas at risk of exposure to enzootic plague are distributed in the east and south of the Plateau. There were 27 species of ticks detected in this region. The tick vectors and possible human infections of Him-TBEV should be urgently evaluated^28^.

## Materials and methods

### Sample collection

The animals of *Marmota himalayana* were sampled in Haixi prefecture (with an altitude of 2994 m above sea level [a.s.l]), in Qinghai province of China in July 2013. A total of 200 marmots were captured by cages in the field and sampled in the laboratory of the Centre for Disease Control and prevention of Haixi prefecture. The sampling was performed in accordance with the protocol for national plague surveillance program in animals. The low respiratory tissue and contents of each animal were separately collected in 2 ml sterile tubes with viral transport medium, which were then stored at −20 ℃ freezer in local laboratory immediately, transported in the same freezer to the laboratory in Beijing. The samples were transferred and storied in −80 °C freezer until use. The study has been reviewed and approved by the ethic committee of National Institute for Communicable Diseases Control and Prevention, China CDC.

### RNA extraction and RNA-seq sequencing

Total RNA of low respiratory sample was extracted using RNeasy Fibrous Tissue Mini Kit (Qiagen, Hilden, Germany).The RNA was eluted in 50 μl RNase-Free Water (Qiagen, Hilden, Germany), which was used for RNA-seq library construction and as template for reverse transcriptase PCR (RT-PCR). The RNAs of low respiratory samples from 100 marmots were randomly chose and merged into 2 pools for RNA-seq library construction and sequencing. After DNase I digestion, total RNA was subjected to an rRNA removal step by using Ribo-Zero Magnetic Gold Kit (Human/Rat/Mouse) and Ribo-Zero Magnetic Gold Kit (Bacterial) (Epicentre, Madison, USA). The remaining RNA was used to construct RNA-seq library according to protocol provided. Briefly, mRNA was purified and enriched using oligo (dT) magnetic beads. The mRNA then was fragmented, and the cDNA was synthesized from the RNA fragments using reverse transcriptase and random primers. After the synthesis of the cDNA, ends repair was performed, followed by adenylation of the 3′ end, ligation of sequencing adapter and quantification using Aglient 2100 Bioanalyzer and ABI StepOnePlus Real-time PCR system. Pair-end (125 bp) sequencing was performed on Hiseq 2500 platform (Illumina, Sandiego, USA). The library construction and sequencing procedures were performed in BGI Tech (Shenzhen, China).

The resulting sequencing reads were trimmed and assembled de novo into contigs using the Trinity software^29^. The assembled contigs were translated and compared to reference protein sequences of all RNA viruses by using local Blastx with an *E*-value of 1e^−5^. The targeted contigs were extracted by a perl script and compared to the entire non-redundant protein database to exclude non-viral sequences using online Blastx. The resulting viral sequences were merged by using SeqMan software within the Lasergene software package version 7.1 (DNAstar, USA) by identifying unassembled overlaps between neighboring contigs.^30^ The newly generated viral sequences were performed a second round of online Blastx and the highly scoring hit was considered as the closet homolog.

### RT-PCR for TBEV detection

For the detection of TBEV between the 200 marmots, a semi-nested RT-PCR method was performed by amplifying a 597-bp fragment spanning the prM-E-coding junction of the virus using specific primers designed based on nucleotide sequences obtained by RNA-seq sequencing.

The first round of semi-nested RT-PCR was performed using PrimeScript One Step RT-PCR kit (TaKaRa, Japan). The RT-PCR mixture (50 μl) contained 2 μl of total RNA, 2 μl PrimeScript one Step Enzyme Mix and 1 μl (20 μM) of each outer primers (F1: 5′-GACTCACTGTCCTATGAGTG-3′, R1:5′-GACCTCCATGACCACTGTGTCAT-3′). After Reverse transcription at 50 °C for 30 min and initial denaturation at 94 °C for 2 min, the mixtures were amplified with 30 cycles of 94 °C for 30 s, 50 °C for 81 s, and 72 °C for 30 s and a final extension at 72 °C for 10 min. Subsequently, 5 μl of the first-round products was used as the template of the second round of PCR amplification. In this round, 20 μl of PCR mixture included 2.5U ExTaq DNA polymerase (TaKaRa, Japan) and 1 μl (20 μM) of each inner primers (F2: 5′-TATGATGCCAACAAGATCGT-3′, R1). After initial denaturation at 94 °C for 5 min, the mixtures were amplified with 30 cycles of 94 °C for 30 s, 50 °C for 36 s, and 72 °C for 30 s and a final extension at 72 °C for 10 min.

Products of second-round PCR were gel purified using a QIAquick gel extraction kit (Qiagen, Hilden, Germany) and sequenced using the ABI prism 3700 DNA Analyzer (Applied Biosystems, Foster City, CA. USA) by Sanger method. The sequences of PCR products were compared with the known sequences of TBEV in the GenBank database.

### Full genome sequencing

The complete genomes of TBEV Himalaya-1 and Himalaya-2 were amplified and sequenced using the RNA extracted from the original sample as templates. The RNA was reverse transcripted and amplified by 11 sets of specific primers (Supplementary Table S3) designed based on multiple alignment of contigs obtained by RNA-seq sequencing and genome sequences of TBEV strains used in Supplementary Table S1. Each amplified segments were sequenced for three times. Genomic Sequences were assembled using SeqMan software (DNAstar, USA).

### Genome analysis

The nucleotide sequences of the genomes and the deduced amino acid sequences of the open reading frame (ORF) were compared to those of other TBEV strains with complete genome. Complete nucleotide sequences of polyprotein and E protein were aligned with clustalW within the BioEdit software (version 7.1). Phylogenetic analysis was conducted using the neighbor-joining method using MEGA 6.0 with 1000 bootstrap replications. Virus strains used in this study were listed in Supplementary Table S2.

### Evolutionary analysis

Complete nucleotide sequences of E protein with known year of collection were used to estimate the mean time of the most recent common ancestor (tMRCA) using the Bayesian Markov Chain Monte Carlo (MCMC) approach employed by BEAST package Version.^31^ The jModeltest software 2.1.7 was used^32^ to estimate the best-fit nucleotide substitution model according to the Akaike information criterion (AIC), with GTR + I + G as the best substitution model. Analyses were conducted under the best-fit nucleotide substitution model and using a relaxed (uncorrelated lognormal) molecular clock model. The MCMC analysis was performed with 20 million generations and was sampled every 1000 generations with 10% burn-in. Convergence of parameters was assessed on the basis of the ESS reaching values >200 by using Tracer software version 1.5. Maximum clade credibility (MCC) trees were subsequently generated after 10% burn-in using Tree Annotator and viewed by FigTree.

## Electronic supplementary material

Supplementary material for revised manuscript
