# Supplementary material for: A new subtype of eastern tick-borne encephalitis virus discovered in Qinghai-Tibet Plateau, China
Source: Emerg Microbes Infect. 2018 Apr 25;7:74. doi: 10.1038/s41426-018-0081-6 (PMC5915441; doi:10.1038/s41426-018-0081-6)
Supplement: Supplementary file 1 — Supplementary material for revised manuscript [file 41426_2018_81_MOESM1_ESM.doc]

Supplementary Table S1 Identities (%) of nucleotide and amino acid sequences of E protein and polyprotein between representative strains of TBEV subtypes

| protein | Him-TBEV* | |  | FE-TBEV | | |  | Sib-TBEV | | |  | Eu-TBEV | | |  | Related flavi | |
| --- | --- | --- | --- | --- | --- | --- | --- | --- | --- | --- | --- | --- | --- | --- | --- | --- | --- |
|  | Him1 | Him2 |  | MDJ | Sofij | 205 |  | Sib | EK | Vasi |  | Neu | Hypr | K23 |  | TSEV | LIV |
| E | | | | | | | | | | | | | | | | | |
| Him1 | × | 99.6 |  | 93.8 | 93.9 | 95.0 |  | 93.8 | 94.2 | 94.2 |  | 93.8 | 92.9 | 93.3 |  | 91.5 | 89.5 |
| Him2 | 99.6 | × |  | 93.3 | 93.5 | 94.6 |  | 93.3 | 93.8 | 93.8 |  | 93.3 | 92.7 | 92.9 |  | 91.1 | 89.1 |
| MDJ | 84.0 | 83.8 |  | × | **99.4** | **98.6** |  | 96.8 | 97.1 | 97.0 |  | 95.6 | 95.2 | 95.2 |  | 92.7 | 91.1 |
| Sofij | 83.9 | 83.8 |  | **93.2** | × | **98.8** |  | 97.0 | 97.4 | 97.2 |  | 95.8 | 95.3 | 95.3 |  | 92.9 | 91.3 |
| 205 | 84.3 | 84.3 |  | **93.2** | **95.5** | × |  | 97.1 | 98.0 | 97.4 |  | 95.8 | 95.4 | 95.4 |  | 92.9 | 91.7 |
| Sib | 83.7 | 83.6 |  | 85.7 | 84.7 | 84.8 |  | × | **98.4** | **98.8** |  | 96.0 | 95.6 | 95.6 |  | 93.3 | 93.1 |
| EK | 84.6 | 84.3 |  | 85.0 | 84.9 | 85.1 |  | **92.7** | × | **98.4** |  | 96.4 | 96.0 | 96.0 |  | 93.7 | 92.3 |
| Vasi | 84.0 | 83.7 |  | 85.7 | 84.7 | 84.6 |  | **95.1** | **93.5** | × |  | 96.2 | 95.8 | 95.8 |  | 93.5 | 92.5 |
| Neud | 83.5 | 83.3 |  | 83.3 | 84.3 | 83.7 |  | 85.1 | 84.9 | 84.9 |  | × | **98.8** | **99.2** |  | 95.8 | 93.8 |
| Hypr | 83.0 | 83.0 |  | 83.4 | 84.2 | 83.6 |  | 85.1 | 84.5 | 84.7 |  | **97.8** | × | **98.4** |  | 95.4 | 93.1 |
| K23 | 82.7 | 82.6 |  | 82.9 | 84.3 | 83.8 |  | 84.9 | 84.6 | 84.6 |  | **97.8** | **97.1** | × |  | 95.4 | 94.0 |
| TSEV | 81.2 | 81.0 |  | 81.6 | 82.7 | 82.1 |  | 82.3 | 82.1 | 82.5 |  | 84.5 | 84.4 | 85.0 |  | × | 93.8 |
| LIV | 81.1 | 81.0 |  | 81.4 | 82.8 | 82.4 |  | 83.1 | 82.6 | 82.9 |  | 86.9 | 87.0 | 87.2 |  | 83.1 | × |
| Polyprotein | | | | | | | | | | | | | | | | | |
| Qh1 | × | 99.8 |  | 93.0 | 93.3 | 93.5 |  | 93.9 | 94.0 | 94.2 |  | 92.9 | 92.6 | 93.0 |  | 91.8 | 90.8 |
| Qh2 | 99.5 | × |  | 92.9 | 93.1 | 93.3 |  | 93.8 | 93.8 | 94.0 |  | 92.8 | 92.6 | 92.8 |  | 91.7 | 90.7 |
| MDJ | 84.1 | 84.0 |  | × | **98.6** | **98.5** |  | 94.8 | 94.9 | 94.7 |  | 93.3 | 93.0 | 93.1 |  | 91.7 | 91.0 |
| Sofij | 84.1 | 84.1 |  | **94.1** | × | **98.6** |  | 95.1 | 95.2 | 95.0 |  | 93.3 | 93.1 | 93.2 |  | 91.8 | 91.3 |
| 205 | 84.5 | 84.4 |  | **94.0** | **95.6** | × |  | 95.0 | 95.2 | 95.1 |  | 93.2 | 93.0 | 93.2 |  | 91.9 | 91.1 |
| Sib | 84.9 | 84.8 |  | 85.2 | 85.5 | 85.2 |  | × | **98.1** | **98.8** |  | 94.3 | 93.8 | 94.1 |  | 93.2 | 92.0 |
| EK | 84.9 | 84.8 |  | 85.0 | 85.4 | 85.2 |  | **93.0** | × | **98.2** |  | 94.3 | 93.9 | 94.1 |  | 93.0 | 92.1 |
| Vasi | 85.2 | 85.1 |  | 85.4 | 85.5 | 85.5 |  | **95.5** | **93.0** | × |  | 94.2 | 93.7 | 94.0 |  | 93.1 | 91.7 |
| Neud | 83.9 | 83.8 |  | 83.3 | 83.4 | 83.2 |  | 84.6 | 84.3 | 84.6 |  | × | **98.8** | **98.9** |  | 93.7 | 94.4 |
| Hypr | 83.8 | 83.7 |  | 83.4 | 83.5 | 83.2 |  | 84.6 | 84.3 | 84.8 |  | **97.2** | × | **98.6** |  | 93.4 | 94.2 |
| K23 | 83.7 | 83.5 |  | 83.2 | 83.3 | 83.2 |  | 84.5 | 84.2 | 84.5 |  | **97.2** | **97.1** | × |  | 93.4 | 94.4 |
| TSEV | 82.4 | 82.3 |  | 82.3 | 82.1 | 82.5 |  | 83.4 | 83.0 | 83.3 |  | 85.3 | 85.1 | 85.3 |  | × | 91.4 |
| LIV | 81.9 | 82.0 |  | 81.7 | 82.2 | 82.1 |  | 83.0 | 83.1 | 83.1 |  | 88.0 | 87.9 | 88.0 |  | 83.4 | × |
| * Abbreviations: Himalaya-1, Him; Himalaya-2, Him2; MDJ-01, MDJ; Sofijin-HO, Sofij; Sib-XJ-X5, Sib; EK-328, EK; Vasichenko, Vasi; Neudoerfl, Neud; Turkish sheep encephalitis virus, TSEV; Louping ill virus, LIV.  Identities of nucleotide sequences are given below the diagonal. Identities of amino acid sequences are given above the diagonal. Identity values within one subtype are shown in boldface. | | | | | | | | | | | | | | | | | |

Supplemental Table S2 Virus strains used in this study

| virus, strain | Accession number | Geographical origin | Year of isolation | Subtype |
| --- | --- | --- | --- | --- |
| TBEV Dalnegorsk | FJ402886 | Dalnegorsk, Russia | 1973 | Far-Eastern |
| TBEV Primorye-739 | JQ825156 | Vladivostok, Russia | 1992 | Far-Eastern |
| TBEV Primorye-87 | JQ825149 | Kavalerovsky, Russia | 1987 | Far-Eastern |
| TBEV Primorye-501 | HQ901367 | Russia | 2010 | Far-Eastern |
| TBEV Primorye-1153 | HQ901366 | Russia | 2009 | Far-Eastern |
| TBEV Primorye-2239 | HM859895 | Russia | 1985 | Far-Eastern |
| TBEV Spassk-72 | JQ825151 | Spassk, Russia | 1972 | Far-Eastern |
| TBEV Primorye-92 | HQ201303 | Vladivostok, Russia | 1992 | Far-Eastern |
| TBEV Primorye-94 | EU816454 | Nadezhdinsky, Russia | 1994 | Far-Eastern |
| TBEV Primorye-91 | JQ825150 | Russia | 1991 | Far-Eastern |
| TBEV Sofjin-Chumakov | KC806252 | Russia | 1937 | Far-Eastern |
| TBEV SofjinKSY | JF819648 | Russia | 1937 | Far-Eastern |
| TBEV 4072 | KF951037 | Russia | 1966 | Far-Eastern |
| TBEV Irkutsk-1861 | JN003205 | Russia | 2008 | Far-Eastern |
| TBEV Chichagovka 1223 | KP844725 | Russia | 2012 | Far-Eastern |
| TBEV Chichagovka 1222 | KP844724 | Russia | 2012 | Far-Eastern |
| TBEV Primorye-633 | HM859894 | Russia | 1978 | Far-Eastern |
| TBEV Primirye-89 | FJ906622 | Arseniev, Russia | 1987 | Far-Eastern |
| TBEV Sofjin-HO | AB062064 | Khabarovsk , Russia | 1937 | Far-Eastern |
| TBEV 1230 | KF880805 | Russia | 2012 | Far-Eastern |
| TBEV Malishevo | KJ744034 | Khabarovsk, Russia | 1978 | Far-Eastern |
| TBEV Khekhtzir 17-13 | KT001072 | Khabarovsk, Russia | 2013 | Far-Eastern |
| TBEV Khekhtzir 10-13 | KT001071 | Khabarovsk, Russia | 2013 | Far-Eastern |
| TBEV Khekhtzir 9-13 | KT001070 | Khabarovsk, Russia | 2013 | Far-Eastern |
| TBEV Birobidzhan 1354 | KP844726 | Russia | 2013 | Far-Eastern |
| TBEV Shkotovo-94 | JQ825147 | Shkotovo, Russia | 1994 | Far-Eastern |
| TBEV Nikolaevsk 855 | KP869172 | Khabarovsk, Russia | 1985 | Far-Eastern |
| TBEV 8696 | KF880804 | Khabarovsk, Russia | 1986 | Far-Eastern |
| TBEV Tomsk-M202 | KJ914683 | Tomsk, Russia | 2008 | Far-Eastern |
| TBEV Tomsk-K6 | KJ739730 | Novosibirsk, Russia | 2008 | Far-Eastern |
| TBEV 205 | DQ989336 | Khabarovsk, Russia | 1973 | Far-Eastern |
| TBEV Tomsk-M83 | KJ739731 | Tomsk, Russia | 2006 | Far-Eastern |
| TBEV Novosibirsk-L2008 | KJ739729 | Novosibirsk, Russia | 2008 | Far-Eastern |
| TBEV Tomsk-PT12 | KM019546 | Tomsk, Russia | 2006 | Far-Eastern |
| TBEV Tomsk-PT14 | KJ914682 | Tomsk, Russia | 2008 | Far-Eastern |
| TBEV 9024 | KF880803 | Amur, Russia | 1990 | Far-Eastern |
| TBEV Oshima 08-As | AB753012 | Hokkaido, Japan | 2008 | Far-Eastern |
| TBEV Oshima 5-10 | AB062063 | Kamiiso, Japan | 1995 | Far-Eastern |
| TBEV Primorye-69 | EU816453 | Ussuriysk, Russia | 2000 | Far-Eastern |
| TBEV Primorye-18 | GQ228395 | Vladivostok, Russia | 1997 | Far-Eastern |
| TBEV Primorye-332 | AY169390 | Nadezhdinsky, Russia | 1991 | Far-Eastern |
| TBEV Primorye-212 | EU816450 | Vladivostok, Russia | 1991 | Far-Eastern |
| TBEV Primorye-253 | EU816451 | Nadezhdinsky, Solovey Kluch, Russia | 1991 | Far-Eastern |
| TBEV Primorye-90 | FJ997899 | Arseniev, Russia | 1990 | Far-Eastern |
| TBEV Primorye-270 | EU816452 | Nadezhdinsky, Mirny, Russia | 1991 | Far-Eastern |
| TBEV Primorye-86 | EU816455 | Kirovsky, Russia | 1986 | Far-Eastern |
| TBEV Primorye-52 | JQ825154 | Shkotovsky, Anisimovka, Russia | 1999 | Far-Eastern |
| TBEV Primorye-196 | JQ825155 | bay Lazurnaya, Russia | 2000 | Far-Eastern |
| TBEV Glubinnoe2004 | DQ862460 | Glubinnoe, Primorsky, Russia | 2004 | Far-Eastern |
| TBEV Lazo MP36 | KT001073 | Lazo, Khabarovsk,  Russia | 2014 | Far-Eastern |
| TBEV Zabaikalye 30-00 | KC422667 | Russia | 2000 | Far-Eastern |
| TBEV Zabaikalye 6-09 | KF826915 | Russia | 2009 | Far-Eastern |
| TBEV Xinjiang-01 | JX534167 | Xingjiang, China | 2012 | Far-Eastern |
| TBEV MDJ01 | JQ650522 | Heilongjiang, China | 2001 | Far-Eastern |
| TBEV WH2012 | KJ755186 | Northern China | 2012 | Far-Eastern |
| TBEV Svetlogorie | GU121642 | Svetlogor’e, Russia | 2008 | Far-Eastern |
| TBEV Kavalerovo | FJ402885 | Kavalerovo, Russia | 1985 | Far-Eastern |
| TBEV Senzhang | JQ650523 | Northeastern China | 1953 | Far-Eastern |
| TBEV MDJ-03 | JF316708 | Heilongjiang, China | 2010 | Far-Eastern |
| TBEV MDJ-02 | JF316707 | Heilongjiang, China | 2010 | Far-Eastern |
| TBEV 178-79 | EF469661 | Irkutsk, Russia | 1979 |  |
| TBEV 886-84 | EF469662 | Irkutsk, Russia | 1984 |  |
| TBEV MGL-Selenge-13-14 | LC017693 | [Mongolia](http://dict.youdao.com/w/Mongolia/#keyfrom=E2Ctranslation) |  | Siberian |
| TBEV Zabaikalye 11-99 | KC414090 | Russia | 1999 | Siberian |
| TBEV Cht-653 | JN003207 | Russia | 1995 | Siberian |
| TBEV Aina | JN003206 | Russia | 1963 | Siberian |
| TBEV Vasilchenko | L40361 | Novosibirsk, Russia | 1969 | Siberian |
| TBEV Irkutsk BR 683-11 | KF823822 | Irkutsk, Russia | 2011 | Siberian |
| TBEV Cht-22 | JN003208 | Russia | 2002 | Siberian |
| TBEV Zabaikalye 1-09 | KF826914 | Russia | 2009 | Siberian |
| TBEV Zabaikalye 68B-00 | KC422663 | Russia | 2000 | Siberian |
| TBEV Irkutsk-12 | JN003209 | Russia | 2010 | Siberian |
| TBEV Sakhalin 6-11 | KF826916 | Russia | 2011 | Siberian |
| TBEV Kolarovo-2008 | FJ968751 | Kolarovo, Russia | 2008 | Siberian |
| TBEV Tomsk-PT122 | KM019545 | Tomsk, Russia | 2006 | Siberian |
| TBEV Sib-XJ-X5 | KP345889 | Xinjiang, China | 2014 | Siberian |
| TBEV Konst-14 | KT321430 | Russia | 2014 | Siberian |
| TBEV Lesopark 11 | KJ701416 | Novosibirsk, Russia | 1986 | Siberian |
| TBEV C11-13 | KP644245 | Novosibirsk, Russia | 2013 | Siberian |
| TBEV Zausaev | AF527415 | Tomsk, Russia | 1985 | Siberian |
| TBEV Buzuuchuk | KJ626343 | Kyrgyzstan | 1986 | Siberian |
| TBEV Latvia-1-96 | GU183382 | Latvia | 1996 | Siberian |
| TBEV Est54 | GU183384 | Estonia | 2000 | Siberian |
| TBEV EK-328 | DQ486861 | Estonia | 1972 | Siberian |
| TBEV Sorex 18-10 | KP938507 | Russia | 2010 | European |
| TBEV IrkutskBR 1456-09 | KP331443 | Russia | 2009 | European |
| TBEV IrkutskBR 1434-09 | KP331442 | Russia | 2009 | European |
| TBEV IrkutskBR 99-08 | KP331441 | Russia | 2008 | European |
| TBEV Vlasaty | KJ922516 | Czech Republic | 1953 | European |
| TBEV Tobrman | KJ922515 | Czech Republic | 1953 | European |
| TBEV Skrivanek | KJ922514 | Czech Republic | 1953 | European |
| TBEV Petracova | KJ922513 | Czech Republic | 1953 | European |
| TBEV Kubinova | KJ922512 | Czech Republic | 1953 | European |
| TBEV Absettarov | KJ000002 | Karelia, Russia | 1951 | European |
| TBEV 285 | KC835596 | Malacky, Slovakia | 1990 | European |
| TBEV 114 | KC835595 | Plastovce, Slovakia | 1980 | European |
| TBEV A104 | KF151173 | Austria | 1990 | European |
| TBEV Absettarov | KU885457 | Leningrad, Russia | 1951 | European |
| TBEV Leila-BH95/15 | KU884607 | Germany | 2015 | European |
| TBEV Toro-2003 | DQ401140 | Toro, Sweden | 2003 | European |
| TBEV KrM 93 | HM535611 | South Korea | 2006 | European |
| TBEV KrM 213 | HM535610 | South Korea | 2006 | European |
| TBEV AS33 | GQ266392 | Amberg, Germany | 2003 | European |
| TBEV Kumlinge | KT224357 | Finland | 1959 | European |
| TBEV Neudoerfl | U27495 | Neudoerfl , Austria | 1971 | European |
| Hypr | U39292 | Brno, Czech Republic | 1953 | European |
| TBEV Kumlinge 25-03 | GU183379 | Finland | 2003 | European |
| TBEV Kumlinge A52 | GU183380 | Finland | 1952 | European |
| TBEV Joutseno | GU183381 | Finland | 1960 | European |
| TBEV Est3476 | GU183383 | Estonia | 2000 | European |
| TBEV Ljubljana I | JQ654701 | Slovenia | 1992 | European |
| TBEV 263 | EU27491 | Temelin,  Czech Republic | 1987 | European |
| TBEV Salem | FJ572210 | Bodensee, Germany | 2006 | European |
| TBEV K23 | AM600965 | Karlsruhe, Germany | 1975 | European |
| SSEV | DQ235152 | Basque region , Spain | 1987 |  |
| LIV 369/T2 | NC_001809 | Scotland,  United Kingdom | 1963 |  |
| TSEV | DQ235151 | Gebze area, Turkey | 1960 |  |
| GGEV | DQ235153 | Vergina, Greece | 1969 |  |

Supplemental Table S3 RT-PCR primers used for amplification of TBEV genome in this study

| Primer ID | Sequence(5’-3’) | Location | Product length |
| --- | --- | --- | --- |
| 5’F | AGATTTTCTTGCACGTGY | 1-17 | 504bp |
| 5’R | CACTCATAGGACAGTGAGTC | 610-629 |  |
| 5’NR | GGTTCTTTCTTTCCGCACTG | 485-504 |  |
| G1F | CAAATGCCAAATGGACTCG | 226-244 | 1204bp |
| G1R | GACCTCCATGACCACTGTGTCATGT | 1938-1962 |  |
| G1NR | CAGCTATGTAGTCCCCCGTGTG | 1408-1429 |  |
| G2F | CTGTCGGACACCAAGGTTGC | 1165-1184 | 1242bp |
| G2R | GATCAAGTTTGTCCACCACC | 2724-2743 |  |
| G2NR | CATGGACATGGTAGGGTTCC | 2387-2406 |  |
| G3F | CTGATAACGCCAAACCCCAC | 2041-2060 | 1563bp |
| G3NF | GAGGTGGATTCATAGAAATG | 2075-2094 |  |
| G3R | CAACACCTCCCCACACAACT | 3618-3637 |  |
| G4F | CTCACRGTGGTGGTGGACAAAC | 2716-2737 | 1532bp |
| G4R | AGTAGGAGGAATGAAGCCACT | 4302-4322 |  |
| G4NR | GTCAGCATGACTCCCACGACAGT | 4225-4247 |  |
| G5F | AGGAAGGACTGGAGTTGGAC | 3960-3979 | 935bp |
| G5R | ATGATTGCCACCTCCCAGTT | 5446-5465 |  |
| G5NR | CCTGCACTGTCTCACCTTTC | 4875-4894 |  |
| G6F | GCTTCGGCTTTTCATTGGTC | 4519-4538 | 1393bp |
| G6R | TGTAGTTACACGCCTGGTCC | 5942-5961 |  |
| G6NR | CTGGCTTGATGTTTGTTCGC | 5892-5911 |  |
| G7F | CCAAGGGTGGTGTCATAGCC | 5714-5733 | 1240bp |
| G7R | GTTTCCAGAGGTCACCAAG | 7687-7705 |  |
| G7NR | GTGGAYAGATCCGCCTTGGTTCTC | 6930-6953 |  |
| G8F | ATTTGGTGTTTCGTCGTTCG | 6664-6683 | 1448bp |
| G8NF | ATGAAATGGGGTTTTTGGAG | 6911-6930 |  |
| G8R | CACAATGTTTCCAGTGACGG | 8339-8358 |  |
| G9F | GCACTACACAGATTCCAACTGC | 8248-8269 | 1734bp |
| G9R | CCACTGGCATGAATGCTCCAAGT | 10048-10070 |  |
| G9NR | GTCACGCCGATGGAAATAGCT | 9961-9981 |  |
| 3’F | GGTTCGGGACAGGTCGTAAC | 9469-9488 | 1129bp |
| 3’NF | CGGAAGAATGCTTGTCAGCG | 9633-9652 |  |
| 3’R | AGCGGGTGTTTTTCCGAGTCAC | 10740-10761 |  |
